# The Use of Contact Tracing Technologies for Infection Prevention and Control Purposes in Nosocomial Settings: A Systematic Literature Review

**DOI:** 10.3390/idr16030039

**Published:** 2024-06-07

**Authors:** Katy Stokes, Davide Piaggio, Francesco De Micco, Marianna Zarro, Anna De Benedictis, Vittoradolfo Tambone, Madison Moon, Alessia Maccaro, Leandro Pecchia

**Affiliations:** 1 Applied Biomedical Signal Processing Intelligent eHealth Laboratory, School of Engineering, University of Warwick, Coventry CV4 7AL, UK; katy.stokes@warwick.ac.uk (K.S.); davide.piaggio@warwick.ac.uk (D.P.); marianna.zarro01@universitadipavia.it (M.Z.); alessia.maccaro@warwick.ac.uk (A.M.); l.pecchia@warwick.ac.uk (L.P.); 2 Research Unit of Bioethics and Humanities, Department of Medicine and Surgery, Università Campus Bio-Medico di Roma, 00128 Roma, Italy; v.tambone@unicampus.it; 3 Department of Clinical Affair, Fondazione Policlinico Universitario Campus Bio-Medico, 00128 Roma, Italy; a.debenedictis@policlinicocampus.it; 4 Research Unit of Nursing Science, Department of Medicine and Surgery, Università Campus Bio-Medico di Roma, 00128 Roma, Italy; 5 Infection Prevention and Control Consultant, Toronto, M4Y 3C8, Canada; mmoon@who.int; 6 Biomedical Engineering (Electronic and Informatics Bioengineering), Università Campus Bio-Medico di Roma, 00128 Roma, Italy

**Keywords:** infection prevention and control, contact tracing, pandemic preparedness, healthcare-associated infections, healthcare risk management, quality of care

## Abstract

Background: Pandemic management and preparedness are more needed than ever before and there is widespread governmental interest in learning from the COVID-19 pandemic in order to ensure the availability of evidence-based Infection Prevention and Control measures. Contact tracing is integral to Infection Prevention and Control, facilitating breaks in the chain of transmission in a targeted way, identifying individuals who have come into contact with an infected person, and providing them with instruction/advice relating to testing, medical advice and/or self-isolation. Aim: This study aims to improve our understanding of the use of contact tracing technologies in healthcare settings. This research seeks to contribute to the field of Infection Prevention and Control by investigating how these technologies can mitigate the spread of nosocomial infections. Ultimately, this study aims to improve the quality and safety of healthcare delivery. Methods: A systematic literature review was conducted, and journal articles investigating the use of contact tracing technologies in healthcare settings were retrieved from databases held on the OvidSP platform between March and September 2022, with no date for a lower limit. Results: In total, 277 studies were retrieved and screened, and 14 studies were finally included in the systematic literature review. Most studies investigated proximity sensing technologies, reporting promising results. However, studies were limited by small sample sizes and confounding factors, revealing contact tracing technologies remain at a nascent stage. Investment in research and development of new testing technologies is necessary to strengthen national and international contact tracing capabilities. Conclusion: This review aims to contribute to those who intend to create robust surveillance systems and implement infectious disease reporting protocols.

## 1. Introduction

On 11 March 2020, COVID-19 was declared a pandemic by the World Health Organization (WHO), only three months after the identification of the first atypical pneumonia case in Wuhan City, China. The COVID-19 etiology was rapidly identified as a strain of coronavirus, later named novel severe acute respiratory syndrome coronavirus 2 (SARS-CoV-2). The clinical presentation of COVID-19 is highly variable with cases that can be managed from home and others that need specialised treatment and intensive care [1]. As of 3 May 2024, there have been over 775 million confirmed cases of COVID-19, including 7 million deaths, according to WHO [2]. Indeed, actual case numbers are likely much greater, as recorded incidences of infection are limited to confirmed cases. In approximately the same time period, a total of 5.47 billion vaccine doses have been administered [2]. Despite previous health threats and emergencies, the world was caught unprepared. Pandemic management was, indeed, as rapid as possible, but the lack of up-to-date preparedness plans had severe social and economic consequences [3,4]. This recent review by Maccaro et al. [5] critically evaluates and reports on the most recent evidence available in the literature related to pandemic preparedness and governance, focusing on principles and practices used during the COVID-19 pandemic. The risk of pandemics is higher than ever before [6] and there is widespread governmental interest in learning from the COVID-19 pandemic in order to ensure the availability of evidence-based Infection Prevention and Control (IPC) measures.

Contact tracing is integral to IPC, facilitating breaks in the chain of transmission in a targeted way. Contact tracing involves identifying individuals who have come into contact with an infected person and providing them with instruction/advice relating to testing, medical advice and/or self-isolation. Various infectious disease outbreaks since the 1980s (e.g., syphilis, AIDS) have driven developments in contract tracing [7]. Implementing contact tracing is complex and relies on trust and community engagement [8]. Little research has focused on contact tracing relative to other public health interventions, in particular focusing on contact tracing in healthcare settings versus community contact tracing. Healthcare settings present a high-risk environment for the spread of infections to vulnerable individuals. This is recognised in the latest WHO guidance on contact tracing, “Contact Tracing and Quarantine in the Context of COVID-19” [9], a document that replaces all previous WHO interim guidance on contact tracing and quarantine in the context of COVID-19 [10,11,12]. This latest guidance highlights that the goal of contact tracing has changed, with the WHO now recommending a risk-based approach, which focuses on priority groups, settings and situations (including healthcare facilities), moving away from the previous intention to interrupt all chains of transmission. In addition, the WHO recommends additional exploration and assessment of digital technologies (e.g., automated proximity tracing, calls and messaging) for contact tracing, as a way to reduce the burden on health systems of running tracing programs.

Conventionally, contact tracing is carried out by specialised healthcare workers (HCWs), who identify contacts based on the infected person’s own recollections. This method was adequate to control previous outbreaks (e.g., SARS-CoV, Ebola), but its efficacy was limited during COVID-19 due to the vastly larger number of cases and the shorter serial interval of transmission [13]. Additionally, traditional contact tracing is inherently time-consuming and requires many HCWs. Technologies provide an opportunity to overcome these obstacles and COVID-19 has driven advancements towards the development of automatised tools for contact tracing. For example, smartphone apps or purpose-built sensors based on Bluetooth, Global Positioning System (GPS) or Wi-Fi signals; however, the legal and ethical implications of using such technologies must be taken into account [14,15].

This project stems from ongoing related work and collaboration with international experts and the WHO (supporting the COVID-19 IPC team of the WHO Emergency Programme—WHE) and between the University of Warwick in the UK and University Campus Bio-Medico in Rome (Italy). As noted in the latest WHO contact tracing guidance, there is a need for a systematic summary of evidence relating to contact tracing technologies [9]. This review seeks to address this gap in the literature. The aim of this study is to improve understanding of the use of contact tracing technologies in healthcare settings, as part of IPC interventions to reduce the spreading of nosocomial infections. This work reviews technology types, performance measures and resources required (including personnel, time, infrastructure, existing servers/computer systems, etc.). It is also noteworthy to mention that this review is in line with another recent review on the use of robots and smart environments for IPC by Piaggio et al. [16]. The information collected is of ongoing use in the aftermath of COVID-19 and aims to help identify priority actions for better preparation for the next global health emergency.

## 2. Methods

### 2.1. Search Strategy and Selection Criteria

The target population, intervention, comparison and outcomes (PICO) of our review are summarised in Table 1.

In order to perform the systematic literature review, relevant keywords were collected via brainstorming during a focus group among biomedical engineers and infection, prevention and control experts. These keywords were enriched with recurrent ones found in relevant scientific articles. The search string included a combination via Boolean operators (e.g., AND, OR) of the following terms and their synonyms: “technology”, “medtech”, “artificial intelligence”, “protocol”, “mobile application”, “mhealth”, “wearable”, “software”, “infection prevention and control”, “transmission”, “pandemic”, “epidemic”, “contact tracing”, “transmission dynamics”, “hospital”, “intensive care unit”, “emergency room” and “surgical theatre”. The full search string can be found in the Supplementary Materials.

The OvidSP database was selected and used to identify relevant journal articles to be included in the review, up to September 2022, with no lower limit date. All the journal articles that were in English language and dealt with contact tracing technologies used within hospital settings for IPC purposes during pandemics/epidemics were retained. Letters to editors, book chapters, editorials, notes and reviews were excluded. Articles that dealt with other technologies for IPC, e.g., sanitation, were excluded. Two of the authors independently assessed the journal articles by title, abstract and, finally, full text, while the third one independently checked the results of the screening, acting as a mediator in case of discordance between the two.

### 2.2. Data Extraction and Quality Appraisal

An ad hoc Excel sheet was prepared for data extraction, and it included information such as title, one-sentence aim of the study, main take-home messages/results, study design (e.g., retrospective study), participants (e.g., patients, healthcare workers, etc.), contact tracing technology (e.g., app, video camera, etc.), performance measure (e.g., accuracy), pathogen (e.g., virus), sources of data (e.g., mobile phones), measurements (e.g., time of contact), hospital department (e.g., emergency department) and comments.

As regards quality appraisal, since the included studies were of mixed type, namely both qualitative and quantitative, it was decided to recur to the use of the MMAT tool [17], which contains different subsections that allow for the assessment of quality of different kinds of studies.

### 2.3. Data Synthesis

The narrative synthesis approach was applied for the synthesis of the collected and extracted data. The salient points in terms of contact tracing technologies and study types were reported and used as the starting point for discussions and potential solution scoping [18].

## 3. Results

### 3.1. Search Strategy and Data Extraction

**Figure 1:**
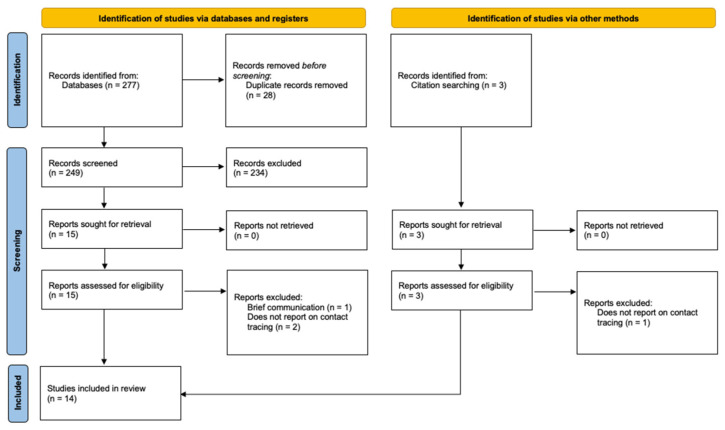
PRISMA flow diagram.

The electronic database (OvidSP) search and study selection process is summarised in Figure 1. The first search was carried out in March 2022. The search returned 243 hits, which were skimmed down to 215 after deduplication. The second search was updated in September 2022. It returned 34 hits, 2 of which were duplicates of the first search. Overall, 14 studies were in line with the inclusion/exclusion criteria and were included in the systematic literature review.

The types of studies are shown in Table 2, most of which described either a retrospective epidemiological analysis or small-scale feasibility or proof of concept investigations. The different technologies, aims, populations and settings among the included studies are presented in Table 3. In total, 7 of the 14 included studies investigated the use of proximity sensing technology [19,20,21,22,23,24,25], 6 reported analyses following the development/deployment of technologically enhanced contact tracing systems [26,27,28,29,30,31], while only 1 described the use of telephone interviews [32]. All studies were conducted in hospital settings, including departments at risk of infectious diseases/isolation rooms [19,23], paediatrics [21,24,28], emergency departments [20,28] and one study included additional data from a nursing home facility [22]. The different technologies used are summarised in Table 4.

Proximity sensing technologies were investigated under different contexts, with some studies addressing understanding of the structure of contacts within certain settings, and others using the generated contact networks to form the basis of epidemiological models. Earlier publications (2011–2013) employed radiofrequency identification (RFID) systems in order to identify contacts based on the proximity of individuals wearing sensors [20,21,23,24]. Lowery-North et al. [20] and Isella et al. [24] determined contact patterns between patients and staff of an emergency department and paediatric ward, respectively, with the aim of further understanding potential transmission routes and the best containment strategies. Lucet et al. used RFID to record interactions between clinical staff and patients over a three-month period [23]. Contacts were compared with observations and interviews to evaluate the accuracy of the RFID-recorded interactions, which were reported to align well. This was the only study that validated the identified contacts. Authors noted that the RFID has the potential for a continuous interaction monitoring system and was well perceived by healthcare workers. Taking a different approach, in 2013, Machens et al. used a dataset containing face-to-face contacts registered by wearable badges enabled with low-power radio communication [21]. Contact patterns were then used to construct and compare simulations of the spread of infectious disease, aiming to inform the construction of epidemiological models with the purpose of identifying high-risk groups and the effectiveness of IPC strategies. More recently (2021), there has been a move away from RFID towards the use of Bluetooth proximity sensors and increased motivation towards the development of automated contact tracing systems [19,22,25]. Curtis et al. performed a pilot study to investigate the use of Bluetooth tags for close contact identification and to compare close contacts in rooms with airborne precautions to those without [19]. Hüttel et al. analysed social interactions identified through a Bluetooth-enabled hand hygiene system in both hospital and nursing home settings [22]. Authors identified medication rooms and kitchens as primary sources of social interaction and therefore areas where IPC should be effective. Further, the epidemiological analysis suggested that quarantine for all the colleagues of a contagious individual is unnecessary. Chambers et al. evaluated a contact tracing solution based on Bluetooth cards, comparing it to self-reported close contacts (standard practice) and to ultra-wide band (UWB) Bluetooth devices [25]. An exit survey showed high acceptability among healthcare workers.

Several studies addressed contact tracing systems targeting specific infectious disease outbreaks. A full contact tracing system, the Surveillance, Outbreak Response Management and Analysis System (SORMAS), was created to stem the West African Ebola virus outbreak of 2014–2015. SORMAS comprises a contact tracing platform accessed through an application for use on smartphones and mobile tablets. Authors report that the majority of users found the tool useful and would recommend its use, with a minority experiencing difficulties in using the platform (specifically logging in). Following the coronavirus outbreak disease in 2019 (COVID-19), three studies report on automated contact tracing approaches [28,29,30]. Hong et al. investigated the use of clinical event data from electronic health records (EHRs) to identify healthcare worker exposure by coronavirus patients. Pairwise HCW–patient interactions were generated automatically based on the EHRs and compared with those identified by the contact tracing team. Interestingly, only 30% of interactions were identified by both systems, with many contacts identified by one system but not the other, and vice versa. Zirbes reports on various development stages of a web-based contact tracing system that was employed during the COVID-19 pandemic from 2020 to 2021. Authors report that moving from a paper-based to a web-based system improved the efficiency and capacity of the system, with contact risk automatically calculated based on an algorithm. Llupià et al. described the SARS-CoV2 Surveillance and Control System (CoSy-19) developed by the Preventive Medicine Department and the Occupational Health Department at Hospital Clinic de Barcelona [30]. The CoSy-19 executes three main tasks: (i) case identification, (ii) contact tracing and (iii) contact follow-up. The system is based on the hospital information system (HIS), a REDCap questionnaire and Go.Data software; however, every case was confirmed by a phone call, making the workflow highly human-dependent. A similar approach was used by Simpson et al. in the context of the global monkeypox outbreak in 2022 [31]. The authors report on the use of a REDCap project that included web-based forms, algorithmic risk classification and symptom-monitoring tools and alerts, making it possible to identify patients for postexposure prophylaxis (PEP).

One study, investigating a COVID-19 outbreak among surgical staff, used only phone call interviews as a contact tracing method; however, Mcdougal et al. performed also environmental sampling and employee mass testing for the purposes of the investigation, leading to uninterrupted surgical service thanks to swift interventions [32].

A single study proposed a whole-genome sequencing (WGS) augmented contact tracing system, addressing the challenges in contact tracing healthcare workers based only on epidemiological data [27]. The authors report that the addition of WGS can help to confirm the suspected intra-hospital spread of severe acute respiratory syndrome coronavirus 2 (SARS-CoV-2), based on the WGS of suspected outbreaks (based on traditional contact tracing) inwards from 10 March 2021 to 1 July 2020.

### 3.2. Quality Appraisal

To assess the quality of evidence from the included studies, the Mixed Methods Appraisal Tool was used. This tool provides a systematic and structured approach to assessing the methodological quality of mixed methods studies. The results of the MMAT quality analysis for each study are provided in the Supplementary Materials. The vast majority of criteria were met by all studies. The most common issue was related to the presence and handling of confounders. In three studies, confounders had not been accounted for in the study design or analysis, and, in a further two, it was unclear. It is good practice to address confounders of studies, as they can result in a biased estimate of the exposure effect by causing the emergence of spurious associations [33,34]. Some difficulty in completing the study as planned arose in Curtis et al., due to issues recruiting volunteers for take-up of proximity sensors; in particular, no medical staff volunteered, and results could only be obtained for nurses. In addition, Lowrey North et al. were not able to distribute the desired proportion of patients with proximity tags, due to the limited capacity of the research team. Furthermore, some Bluetooth cards’ data were lost due to firmware errors in Chambers et al.’s study.

## 4. Discussion

Healthcare settings are high-risk environments for infectious disease outbreaks. The COVID-19 pandemic revealed facilities to be poorly prepared to respond to the challenges of novel pathogens, particularly relating to the tracing of infections [3,5,35,36]. The use of rapid diagnostic tests and contact tracing technologies in healthcare settings is a key response to future pandemic threats, as well as addressing the silent burden of healthcare-associated infections (HAI) [37,38].

To deepen the understanding in this area, this systematic review of the literature was designed to gather evidence on the use of contact tracing technologies in healthcare settings, specifically, to map the existing technologies and to assess their effectiveness.

There was a high level of heterogeneity among the included studies, and no two studies investigated the same contact tracing approaches or measured the same outcomes. The majority of studies presented findings from highly specific and small-scale pilot studies for which the generalisation of findings is challenging. Additionally, controls or comparisons with gold standard or confirmed cases were not available in most studies, with only one study able to validate the identified contacts. Perceptions from healthcare facility staff were positive. Overall, this highlights the relatively nascent stage in this field of research. Despite these challenges, included studies reported positive findings, supporting further research and wider investigations.

Fifty percent of the studies examined proximity detection technologies using either radio frequency identification (RFID) systems [28,29,31,32] or Bluetooth proximity sensors [27,30,33]. These systems allow monitoring of the location and movements of healthcare personnel within healthcare facilities. This can be particularly useful in the context of healthcare-associated infections (HAIs), as it enables rapid identification of healthcare workers (HWs) who had contact with infected patients and taking necessary measures to limit the spread of infections within healthcare facilities [39].

Furthermore, contact tracing technologies in healthcare settings contribute to identifying areas for improvement in HAI prevention practices, optimising the organization of activities and distributing resources. The collected data can be analysed to identify patterns of movement among healthcare personnel, equipment and patients. They can support the training and education of healthcare staff, thereby improving their skills and awareness regarding HAI prevention practices. Additionally, the data can provide insights for evaluating the effectiveness of preventive measures implemented in healthcare facilities, such as proper use of personal protective equipment (PPE) or compliance with hand hygiene procedures [16,39,40].

Both RFID systems and Bluetooth proximity sensors showed high acceptability among healthcare providers [31]. However, it is crucial to ensure that data sharing occurs while respecting privacy and patients’ safety. Privacy and data security are paramount in healthcare data management. It is imperative to ensure informed consent from patients regarding the collection, storage and use of their data. Additionally, adequate security measures must be implemented to protect data from unauthorised access, loss, or breaches. The privacy of HWs should also be safeguarded, as the use of these devices could potentially lead to the monitoring of workflow and personnel [41,42]. According to Articles 13 and 14 of the GDPR, every EU citizen has the right to be informed about the processing of their personal data [43]. This becomes even more complex when taking into account artificial intelligence-based systems, which are interwoven with further ethical challenges [44]. A different approach involves the use of technologies for extracting information from healthcare documentation. This has allowed for the identification of HWs’ exposure to patients with infectious pathogens, as well as the identification of cases, contact tracing and follow-up [45,46]. The information in EHRs allows for the identification and monitoring of high-risk patients, such as those with compromised immune systems or who have undergone invasive procedures [47]. By analysing the data collected from EHRs, the most common types of infections, high-risk departments or areas within the hospital, as well as patients who may be more susceptible, can be identified. This information is crucial for the development of targeted prevention strategies and the implementation of specific control measures [48]. Furthermore, EHRs can be integrated with epidemiological surveillance systems to quickly identify adverse events, collect data on the frequency and severity of infections and monitor the effectiveness of implemented control measures. This integration allows for enhanced surveillance capabilities and timely response to emerging issues.

In a single study, telephone interviews were used as a contact tracing method to investigate a COVID-19 outbreak among surgical staff [49]. Telephone interviews provide several advantages in contact tracing, such as reaching a large number of people quickly and collecting detailed and accurate information. Interviewers can ask specific questions about symptoms, frequented locations and close contacts of infected individuals. These details are crucial for identifying potential infections and taking timely measures to prevent the spread of the infection. However, telephone interviews may have some limitations. Some individuals may forget or underestimate the details of their contacts, or they may not have access to a phone and people may not be available to be contacted. These factors can compromise the effectiveness of contact tracing [50,51].

Finally, a study proposed an enhanced contact tracing system with whole-genome sequencing (WGS) [52]. Genome sequencing enables the analysis of the entire DNA of an organism, providing a comprehensive view of the genetics of infectious diseases [53]. This technology allows for the analysis of genetic sequences from viral or bacterial strains isolated from infected patients and comparing them to identify similarities and differences [27,52,54]. Contact tracing through WGS can have a significant impact on public health surveillance as it can help identify relationships between cases and reconstruct the transmission pathways of diseases. Additionally, WGS can enable health authorities to identify “super-spreaders”, individuals who have a high capacity to transmit the disease to others. By identifying these individuals, targeted preventive measures such as isolation or closer monitoring can be implemented to reduce the spread of the disease [55,56].

The development of contact tracing through advanced technological systems could help healthcare systems around the world meet the WHO’s proposed goals to improve patient safety. According to the WHO, it is crucial to implement rigorous evidence-based measures to prevent and control infections, reducing HAIs and antimicrobial resistance. There is a need to develop surveillance systems for these infections and to ensure adequate laboratory testing capabilities at local, national and global levels to improve detection and response to multi-drug-resistant organisms in healthcare settings [57]. The tracking of contacts using RFID systems [25,26,28,29] or Bluetooth proximity sensors [24,27,30], and the extraction of information from healthcare records, are all useful resources to identify healthcare workers and mitigate the risk of spreading infections within a hospital. Furthermore, they are valuable tools for improving prevention practices and monitoring improvement actions.

Certainly, the costs associated with contact tracing through genome sequencing can vary depending on various factors, including the scale of the epidemic, the volume of samples to be sequenced and the available technological resources. Sequencing a large number of samples can require sophisticated and expensive equipment, qualified and trained personnel and appropriate laboratory infrastructure. This raises a public health ethical issue that should not be overlooked. The benefits of using contact technologies for epidemiological control could exclude populations living in rural areas and with difficult access to technology, potentially exacerbating already existing health inequalities [58,59]. This phenomenon, called the ‘digital divide’, refers to the disparity between individuals, households, businesses, and geographic areas with regard to access, use and skills in the use of digital technologies. Inequality can disproportionately affect marginalised communities and frail patients [60,61]. The ethical consequences of the digital divide need to be addressed; otherwise, there is a real risk that advanced technology, which could potentially improve people’s health, paradoxically becomes a means of discrimination and widens inequalities [62].

## 5. Conclusions

Pandemics are a known risk, and the lack of preparedness and inadequate emergency plans have amplified the devastating impact of the COVID-19 pandemic. It is mandatory to learn from this crisis and take measures to ensure better preparedness and a more effective response in the future. This work aimed to assess the academic literature relating to the use of contact tracing technologies in healthcare settings, and their effectiveness in limiting the spread of infection. Studies reported both promising performance and good acceptability of contact tracing technologies. Published research to date comprises early-stage feasibility and proof of concept research. In order to build on these foundations, investment in research and development of new testing technologies is necessary. Ultimately, these technologies may be used to strengthen national and international contact tracing capabilities. This review contributes insights relevant to researchers and developers seeking to create robust surveillance systems for reducing the transmission of infections in healthcare settings.

## Figures and Tables

**Table 1:** The Problem, Intervention, Comparison and Outcomes (PICO) framework used for the structured formulation of the review questions.

**Population**	Healthcare-acquired infections
**Intervention**	Technologies for contact tracing in healthcare settings for infectious disease
**Comparison/control**	Current standard, or may be no comparison
**Outcome**	(1) What? (2) Performance measures (3) Costs and resource needs (including personnel, time, infrastructure, existing servers/computer systems, etc.)

**Table 2:** Study types.

Study Type	Number of Studies
Epidemiological analysis following outbreak	6
Feasibility/proof of concept	7
Evaluation of user perceptions	1

**Table 3:** Technologies, aims, populations and settings among the included studies.

	Technology	Objective	Population	Study Setting	Outbreak of Interest
Tom-Aba et al., 2018 [26]	Web and mobile application	User perceptions of system	Clinical staff	Hospital, Nigeria	Various
Løvestad et al., 2021 [27]	Nanopore whole-genome sequencing	Evaluation of a sequencing complemented contact tracing system	Clinical staff (24) and patients (2)	Several hospital wards	COVID-19
Curtis et al., 2022 [19]	Bluetooth wearable proximity sensors and gateway system for data collection and cloud upload	Evaluation of the feasibility of proximity sensing to generate contact networks	Clinical staff: nurses (27) and doctors	Negative-pressure hospital isolation rooms	General
Hong et al., 2021 [28]	Automated electronic health record analysis system	Evaluation of performance of automated system to identify contacts	Hospital staff: clinical and non-clinical and patients (211)	Paediatric hospital (quaternary care), including inpatient, outpatient and emergency department	COVID-19
Zirbes et al., 2021 [29]	Automated system including web-based forms and algorithmic risk classification and alerts	Report on the design and usage of their contact tracing workflow for identifying healthcare worker exposures	Clinical staff and patients with COVID-19 (233)	Hospital	COVID-19
Lowery-North et al., 2013 [20]	Radiofrequency identification (RFID) system including wearable proximity sensors	Evaluation of contact characteristics using automated system	Clinical staff (88) and patients (4732)	Emergency department	General
Machens et al., 2013 [21]	Wearable proximity sensors	Comparison of numerical simulations to identify spread of infectious disease	Hospital staff: physicians (20), nurses (21), ward assistants (10), caregivers (31) and patients (37)	Hospital, paediatric ward	H1N1
Hüttel et al., 2021 [22]	Bluetooth wearable proximity sensors	Evaluation of the social interactions occurring in healthcare settings	Hospital staff: nurses (123), doctors (86), cleaning staff (11)	Hospital and nursing home facility	COVID-19
Lucet et al., 2012 [23]	Radiofrequency identification (RFID) system including wearable proximity sensors	Evaluation of automatic system for recording healthcare working and patient interactions	Clinical staff (82) and patients (54)	Two clinical wards in infectious diseases department, pulmonology unit with patients under ‘airborne precautions’	Tuberculosis
Isella et al., 2011 [24]	Radiofrequency identification (RFID) system including wearable proximity sensors	Evaluation of automatic recording of healthcare worker and patient interaction	Clinical staff (51), patients (37) and caregivers (31)	Hospital, general paediatric ward	General
Simpson et al., 2022 [31]	Semi-automated system including web-based forms, algorithmic risk classification, symptom-monitoring tool and alerts	Report on the design and usage of contact tracing and symptoms monitoring workflow	Healthcare personnel with possible exposure to monkeypox	Hospital	Monkeypox
McDougal et al., 2022 [32]	Mass testing and phone call interviews	Report on outbreak investigation using contact and environmental sampling	Operating room staff with positive SARS-CoV-2 molecular testing (24)	Hospital, OR	COVID-19
Llupià et al., 2022 [30]	Semi-automated system including web-based forms and chain of transmission identification	Report on the design and usage of contact tracing workflow to assess the impact on the number of contacts that new cases generated	Patients (1980 + 443), Heath care workers (384 + 1341)	Hospital	COVID-19
Chambers et al., 2022 [25]	Bluetooth proximity sensor	Evaluate contact tracing solution, in particular, Bluetooth-enabled card to identify contacts	Hospital staff: Nurse (18), Junior Doctor (9), Allied Health Team Member (5), Senior Doctor (4), Ward Administrative Staff (3), Ward Domestic Staff (2), Other (CNM) (1)	Hospital	COVID-19

**Table 4:** Technologies employed.

Technology	Number of Studies
Proximity sensing	7
Web/mobile application	4
Automated health record analysis	1
Whole genome sequencing complemented system	1
Interview	1

## Data Availability

The data presented in this study are available upon reasonable request from the authors of this manuscript.

## Supplementary Materials

The following supporting information can be downloaded at: https://www.mdpi.com/article/10.3390/idr16030039/s1.
