# Supplementary material for: The Use of Contact Tracing Technologies for Infection Prevention and Control Purposes in Nosocomial Settings: A Systematic Literature Review
Source: Infect Dis Rep. 2024 Jun 7;16(3):519–30. doi: 10.3390/idr16030039 (PMC11203438; doi:10.3390/idr16030039)
Supplement: Supplementary file 1 [file idr-16-00039-s001.zip › idr-2944838-supplementary.pdf]

# Search string

(technolog* or medtech* or medical technolog* or healthtech* or health technolog* or device or AI or artificial intelligence or deep learning or machine learning or protocol or digital or mobile application* or mobile app* or smartphone* or smart phone* or ICT* or mhealth or ehealth or wearable* or software).mp. [mp=ab, td, hw, ti, tx, bt, ct, sh, tn, ot, dm, mf, dv, kf, fx, dq, an, ui, jn, ja, tt, nm, ox, px, rx, ds, on, sy, tc, id, tm]

AND

((Infection prevention and control) or IPC or infection prevention or infection control or transmission control or pandemic or epidemic or outbreak).mp. [mp=ab, td, hw, ti, tx, bt, ct, sh, tn, ot, dm, mf, dv, kf, fx, dq, an, ui, jn, ja, tt, nm, ox, px, rx, ds, on, sy, tc, id, tm]

AND

(contact trac* or transmission dynamics).mp. [mp=ab, hw, ti, tx, bt, ct, tn, ot, dm, mf, dv, kf, fx, dq, an, jn, tt, nm, ox, px, rx, ui, sy, tc, id, tm, td]

AND

(hospital or ICU or intensive care unit or clinic or infirmary or healthcare setting or health center or healthcare location or health centre or healthcare setting or health location or emergency cent* or emergency room or ER or "accident and emergency" or "A and E" or "A&E" or nosocom* or ((surg* or operat*) and (room or theatre or suite)))

# Full texts excluded with reasons

| First Author | Title | Reason for exclusion |
|---|---|---|
| Wee | Utilisation of SARS-CoV-2 rapid antigen assays in screening asymptomatic hospital visitors: mitigating the risk in low-incidence settings. | Evaluating antigen testing, not contact tracing |
| Fillmore | An application to support COVID-19 occupational health and patient tracking at a Veterans Affairs medical center | Brief communication |
| Hornbeck | Using Sensor Networks to Study the Effect of Peripatetic Healthcare Workers on the Spread of Hospital-Associated Infections | Not reporting on contact tracing |
| Hare | Repeated transmission of SARS-CoV-2 in an overcrowded Irish emergency department elucidated by whole-genome sequencing. | Not reporting on contact tracing, but focusing on outbreak description |

# MMAT results

| First author | Year | Citation | SCREENING QUESTIONS S1. Are there clear research questions? | S2. Do the collected data allow to address the research questions? | 1. QUALITATIVE STUDIES 1.1. Is the qualitative approach appropriate to answer the research question? | 1.2. Are the qualitative data collection methods adequate to address the research question? | 1.3. Are the findings adequately derived from the data? | 1.4. Is the interpretation of results sufficiently substantiated by data? | 1.5. Is there coherence between qualitative data sources, collection, analysis and interpretation? | 2. RANDOMIZED CONTROLLED TRIALS 2.1. Is randomization appropriately performed? | 2.2. Are the groups comparable at baseline? | 2.3. Are there complete outcome data? | 2.4. Are outcome assessors blinded to the intervention provided? | 2.5 Did the participants adhere to the assigned intervention? | 3. NON-RANDOMIZED STUDIES 3.1. Are the participants representative of the target population? | 3.2. Are measurements appropriate regarding both the outcome and intervention (or exposure)? | 3.3. Are there complete outcome data? | 3.4. Are the confounders accounted for in the design and analysis? | 3.5. During the study period, is the intervention administered (or exposure occurred) as intended? | 4. QUANTITATIVE DESCRIPTIVE STUDIES 4.1. Is the sampling strategy relevant to address the research question? | 4.2. Is the sample representative of the target population? | 4.3. Are the measurements appropriate? | 4.4. Is the risk of nonresponse bias low? | 4.5. Is the statistical analysis appropriate to answer the research question? |
|---|---|---|---|---|---|---|---|---|---|---|---|---|---|---|---|---|---|---|---|---|---|---|---|---|
| Tom-Aba | 2018 | User Evaluation Indicates High Quality of the surveillance outbreak response management and analysis system after field deployment in Nigeria in 2015 and 2018 | Yes | Yes | Yes | Yes | Yes | Yes | Yes | | | | | | | | | | | | | | | |
| Lovestad | 2021 | Investigation of intra-hospital SARS-CoV-2 transmission using nanopore whole-genome sequencing. | Yes | Yes | | | | | | Yes | Yes | Yes | Yes | Yes | | | | | | | | | | |
| Curtis | 2022 | Feasibility of Bluetooth Low Energy wearable tags to quantify healthcare worker proximity networks and patient close contact: A pilot study. | Yes | Yes | | | | | | Yes | Yes | No | No | No | | | | | | | | | | |
| Hong | 10 | Use of clinical data to augment healthcare worker contact tracing during the COVID-19 pandemic. | Yes | Yes | | | | | | Yes | Yes | Yes | Yes | Yes | | | | | | | | | | |
| Zirbes | 2021 | Development of a web-based contact tracing and point-of-care-testing workflow for SARS-CoV-2 at a German University Hospital. | Yes | Yes | | | | | | Yes | Yes | Yes | Yes | Yes | | | | | | | | | | |
| Lowery-North | 2013 | Measuring social contacts in the emergency department. | Yes | Yes | | | | | | Yes | Yes | Yes | Yes | No | | | | | | | | | | |
| Machens | 2013 | An infectious disease model on empirical networks of human contact: bridging the gap between dynamic network data and contact matrices. | Yes | Yes | | | | | | Yes | Yes | Yes | Yes | Yes | | | | | | | | | | |
| Hüttel | 2021 | Analysis of social interactions and risk factors relevant to the spread of infectious diseases at hospitals and nursing homes | Yes | Yes | | | | | | Yes | Yes | Yes | No | Yes | | | | | | | | | | |
| Lucet | 2012 | Electronic Sensors for Assessing Interactions between Healthcare Workers and Patients under Airborne Precautions | Yes | Yes | | | | | | Yes | Yes | Yes | Yes | Yes | | | | | | | | | | |
| Isella | 2011 | Close Encounters in a Pediatric Ward: Measuring Face-to-Face Proximity and Mixing Patterns with Wearable Sensors | Yes | Yes | | | | | | Yes | Yes | Yes | Unclear | Yes | | | | | | | | | | |
| Simpson | 2022 | Development and deployment of tools for rapid response notification of Monkeypox exposure, exposure risk assessment and stratification, and symptom monitoring. | Yes | Yes | Yes | Unclear | Unclear | Unclear | Unclear | | | | | | | | | | | | | | | |
| McDougal | 2022 | Outbreak of coronavirus disease 2019 (COVID-19) among operating room staff of a tertiary referral center: An epidemiologic and environmental investigation. | Yes | Yes | | | | | | | | | | | | | | | | Yes | Yes | Unclear | Yes | Yes |
| Llupià | 2022 | SARS-CoV2 hospital surveillance and control system with contact tracing for patients and health care workers at a large reference hospital in Spain during the first wave: An observational descriptive study. | Yes | Yes | | | | | | | | | | | | | | | | Yes | Yes | Yes | Unclear | Yes |
| Chambers | 2022 | Pilot of a digital contact tracing card in a hospital setting in New Zealand, 2020. | Yes | Yes | | | | | | | | | | | Yes | Yes | Yes | No | Yes | | | | | |

| | |
|---|---|
| ● | Unclear |
| ● | Yes |
| ● | No |
